# Facile and scalable tubing-free sample loading for droplet microfluidics

**DOI:** 10.1038/s41598-022-17352-3

**Published:** 2022-08-03

**Authors:** Fangchi Shao, Kuangwen Hsieh, Pengfei Zhang, Aniruddha M. Kaushik, Tza-Huei Wang

**Affiliations:** 1 Department of Biomedical Engineering, Johns Hopkins University, 3400 N. Charles Street, Baltimore, MD 21218 USA; 2 Department of Mechanical Engineering, Johns Hopkins University, 3400 N. Charles Street, Baltimore, MD 21218 USA

**Keywords:** Lab-on-a-chip, Techniques and instrumentation, Characterization and analytical techniques, Nanofluidics

## Abstract

Droplet microfluidics has in recent years found a wide range of analytical and bioanalytical applications. In droplet microfluidics, the samples that are discretized into droplets within the devices are predominantly loaded through tubings, but such tubing-based sample loading has drawbacks such as limited scalability for processing many samples, difficulty for automation, and sample wastage. While advances in autosamplers have alleviated some of these drawbacks, sample loading that can instead obviate tubings offers a potentially promising alternative but has been underexplored. To fill the gap, we introduce herein a droplet device that features a new Tubing Eliminated Sample Loading Interface (TESLI). TESLI integrates a network of programmable pneumatic microvalves that regulate vacuum and pressure sources so that successive sub-microliter samples can be directly spotted onto the open-to-atmosphere TESLI inlet, vacuumed into the device, and pressurized into nanoliter droplets within the device with minimal wastage. The same vacuum and pressure regulation also endows TESLI with cleaning and sample switching capabilities, thus enabling scalable processing of many samples in succession. Moreover, we implement a pair of TESLIs in our device to parallelize and alternate their operation as means to minimizing idle time. For demonstration, we use our device to successively process 44 samples into droplets—a number that can further scale. Our results demonstrate the feasibility of tubing-free sample loading and a promising approach for advancing droplet microfluidics.

## Introduction

Recent years have witnessed the emergence of droplet microfluidics as enabling tools in a wide range of analytical and bioanalytical research^1–3^ from chemical synthesis^4,5^, directed evolution^6,7^, high-throughput drug screening^8^, green chemistry and nanoparticle fabrication^9^, crystallization^10^, diagnosis^11^, to single-molecule and single-cell analysis^12^. Indeed, by discretizing a single bulk-based reaction into massive numbers of nano- to picoliter droplets that can all function as reaction compartments, droplet microfluidics offers compelling advantages in detection sensitivity, assay time, reagent consumption, and cost^1,13^. As such, advancing droplet microfluidics has become a burgeoning research area. Most of the research efforts in this area to date have focused on droplet generation^14–18^ and droplet handling (e.g. mixing, trapping, splitting, incubating, and releasing)^19–24^. Despite these advances, sample loading in droplet microfluidics continues to rely on tubing-based interfaces^25–27^. That is, samples are injected through designated tubings and inlets into droplet devices via pressure sources such as syringe pumps. Although tubing-based interfaces offer practical means for loading one to several samples, they face challenges when tens, hundreds, or even more samples are to be analyzed (a common scenario in analytical and bioanalytical applications) and have additional drawbacks such as difficulty for automation and sample wastage. Therefore, besides droplet generation and droplet handling, improvements to sample loading are also critical for advancing droplet microfluidics.

In principle, there are three approaches for improving sample loading for droplet microfluidics. One approach is retaining tubings for loading samples into droplet microfluidic devices and improving external instrumentation. To this end, autosamplers are emerging solutions with promises of scalability and automation^28–36^. Autosamplers typically store multiple samples and leverage various sample switching mechanisms (e.g., motorized stage^28,35,36^, rotational stage^29^, high-performance liquid chromatography^30^, injection loops^31^) to load multiple samples through a single tubing to the droplet microfluidic device. The second approach replaces tubings with more convenient reservoirs and tanks. However, despite some research advances^17,37–39^ and commercialization (e.g., commercial foundries such as Microfluidic Chip Shop), each reservoir or tank typically still holds only one sample and thus still lacks scalability. The third approach is completely freeing droplet microfluidic devices from tubings, reservoirs, or tanks during sample loading, thereby lifting their imposed restrictions to scalability. Unfortunately, despite its potential appeal, such a sample loading approach has yet to be demonstrated. Consequently, the potential of this approach for enhancing droplet microfluidics—including in scalability, sample wastage, automation, and idle time—remains unexplored.

In response, we have developed a novel Tubing Eliminated Sample Loading Interface (TESLI) and have incorporated it within an integrated droplet device. TESLI integrates a network of programmable pneumatic microvalves that regulate vacuum and pressure sources so that successive sub-microliter samples can be directly spotted onto the open-to-atmosphere TESLI inlet, infused into the device via vacuum, and partitioned into nanoliter droplets within the device via pressure with minimal wastage. Before the next sample is spotted, infused, and partitioned into droplets, TESLI performs simple and effective cleaning to alleviate cross-contamination. Moreover, to minimize idle time, we have implemented two TESLIs in our device to parallelize and alternate their operation. Additionally, we incorporated droplet incubation and fluorescence-based droplet detection in our device, thereby facilitating a streamlined workflow from samples to analyses. For demonstration, we used this integrated droplet device with dual TESLIs to process and analyze 44 samples—a number that can further scale—with only nanoliter sample wastage and zero idle time, while displaying strong potential for full automation.

## Experimental section

### Device design

The microfluidics device is composed of two PDMS layers with a fluidics layer on top for droplet generation, incubation, and detection and a valve layer at the bottom that controls of the actuation of the microvalves. The fluidics layer is designed with 11 distinct inlet and outlet ports, including two sample inlets, one pressure inlet, one vacuum outlet, two oil inlets, two pressure-release outlets, two reagent inlets, and one sample outlet. The droplet assembly are taken place at a channel with width of 200 μm to help squeeze the droplet for better merging of different reagents and samples. A mixing channel with serpentine shape is subsequently placed after the droplet assembly channel to help the mixing of different chemical or biological contents in the droplets. Immediately downstream the mixing channel, the channel gradually widens and connects to a serpentine incubation channel that is 500 μm wide and 50 cm long. The end of the incubation channels is narrowed into a 100-μm-wide detection constriction, where the detected droplets eventually flow into the device outlet port. The bottom valve layer consists of 13 ports that control the actuation of 13 corresponding microvalves. Among the 13 microvalves, one microvalve controls the droplet assembly oil, one microvalve controls the surfactant oil, two microvalves control the vacuum outlet, two microvalves control the pressure inlet, one microvalve controls the pressure releasing outlets, two microvalves control the sample loading inlets, and four microvalves control the assembly of the samples and reagents.

### Master mold fabrication

The photomasks for both PDMS layers were designed using AutoCAD 2020 (Autodesk, San Rafael, CA, USA) and the mask patterns were printed onto high precision transparencies at 20,000 dpi by CAD/Art Services Inc. (Bandon, OR, USA). The master mold for both fluidics layer and valve layer were fabricated using standard photolithography on 4-inch silicon wafers (Polishing Corporation of America, Santa Clara, CA, USA). To fabricate the mold for the fluidics layer, SPR-220-7 (positive photoresist; Microchem Corp., Newton, MA, USA) was initially spin-coated onto the wafer with a height of 35 μm, which served as the segment to interact with the bottom valve layer due to the rounded cross-section of SPR-220-7 after hard baking. The push-up valve architecture of the device allows the bottom valve layer to collapse into the top fluidics layer patterned with SPR-220-7 to achieve tight valve sealing or closing. The remaining fluidic channels that were not interacting with microvalves were fabricated by spin-coating additional layer of SU8-3025 (negative photoresist; Microchem Corp., Newton, MA, USA) onto the silicon wafer with a height of 50 μm. The SU-8 and SPR-220-7 channels were aligned with mask aligner using the predesigned alignment markers. The mold of the valve control layer was patterned with single layer of SU8-3025 (negative photoresist; Microchem Corp., Newton, MA, USA) with a height of 30 μm via standard photolithography.

### Microfluidics device fabrication

The device was fabricated with polydimethylsiloxane (PDMS) using multilayer soft lithography. Both molds were firstly silanized with chlorotrimethylsilane (Sigma-Aldrich, St. Louis, MO, USA) in the desiccator for 10 min to reduce the adhesion between the PDMS and the photoresist on the molds. The thin valve layer was obtained by spin coating PDMS (SYLGARD 184 Silicone Elastomer Kit, Dow Corning, Midland, MI, USA) with 15:1 base to curing agent ratio on the mold of the valve control layer at 1000 rpm and subsequently curing for 20 min at 80 °C. The thick fluidics layer was prepared by pouring 51.7 g of 10:1 (PDMS base to curing agent ratio) on the fluidics mold and baking for 20 min at 80 °C. After the baking of both molds, the cured PDMS on the fluidics mold was peeled off while the cured PDMS on the valve control mold were retained. The peeled PDMS and the PDMS coated valve control mold were then manually aligned and bonded under Stemi DV4 Microscopy (Carl Zeiss AG, Oberkochen, Germany) followed with oxygen plasma treatment (42 W, 500mTorr, 45 s). After 5 min post-baking at 80 °C, the fluidics PDMS layer and the valve control PDMS layer were permanently bonded and the bonded double-layer PDMS was peeled off for punching holes through all the ports. The double-layer PDMS was then permanently bonded with 48 × 65 mm cover glass (Thermo Fisher Scientific, Waltham, MA, USA) with oxygen plasma treatment (42 W, 500mTorr, 45 s) and 5-min post baking at 80 °C. The fabricated microfluidic device was then stored in the oven for ~ 48 h at 80 °C before the operation.

### Device operation

In our integrated droplet device, nanoliter droplets were assembled by programmatically actuating the microvalves that were interacted with a set of solenoid valves controlled by custom codes written in MATLAB (MathWorks, Natick, MA, USA). The interface between the microvalves and the solenoid valves were water-filled Tygon microbore tubing (0.02-inch ID and 0.06-inch OD; Cole-Parmer, Vernon Hills, IL, USA) connected to 23-gauge blunt needles (McMaster-Carr), which were inserted at the designated valve inlet ports. Pressure was set to be 30 psi to ensure fully closed valves during the operation to avoid the cross talk. By controlling the opening and closing of the microvalves that regulated the sample and reagent inlets, droplets with desired composition and size were able to be generated. The oil for droplet assembly was composed of fluorinated oil FC-40 (3M, Two Harbors, MN, USA) and nonionic fluorous-soluble surfactant 1H, 1H, 2H, 2H-perfluoro-1-octanol (PFO; Sigma-Aldrich) with a ratio of 4:1. We used the droplet generation oil for EvaGreen (BIO-RAD, Hercules, CA, USA) as the surfactant oil to pre-treat the incubation channel to avoid droplet sticking onto the channel wall. Both oils were pre-loaded in the Tygon microbore tubing and connected to the oil inlet ports with 23-gauge blunt needles. A simple vacuum trap was fabricated with one microcentrifuge tube (Eppendorf North America, CT, USA) and two Tygon microbore tubing as the bridge between the microfluidic device and the vacuum source, which served the role of storing all the wastes and avoiding the direct communication with the vacuum source. The microcentrifuge tube was firstly drilled with two holes for inserting the tubing, with one inserted all the way to the bottom and one inserted near the cap. An epoxy glue (3M, Saint Paul, MN, USA) was used to seal the gap between the tubing and the holes to ensure the vacuum environment in the microcentrifuge tube. The pressure input for all the inlets of the device were optimized and kept the same with 5 psi for reagent inlets, and 3.5 psi for droplet assembly oil and sample inlets.

Before the operation, the surfactant oil was first injected to fill the device to pre-treat the incubation channels, which was later pushed out and refilled by droplet assembly oil. During the operation, each sample (~ 0.7 μL) was directly pipetted onto the device inlet while the TESLI activated the vacuum to draw the sample into the sample storage channels and wait for further instructions. DI water functioning as the wash buffer was also pipetted onto the sample device inlet port by following the pre-designed queues. With the programmed instructions, droplet assembly, incubation, and detection were achieved automatically.

### Evaluation of cleaning efficiency and dead volume

The cleaning efficiencies of three cleaning protocols were calculated by obtaining the fluorescence images of the sample storage channels during the operation at different time points. We purchased FAM green fluorescence dye from Thermo Fisher Scientific (Waltham, MA, USA) and diluted it to 125 nM as the input sample. The images of the channels were captured by the digital single-lens reflex (DSLR) camera (EOS 60D; Canon, Inc., Tokyo, Japan) that was mounted on the fluorescence microscopy (Olympus IX71, Shinjuku, Tokyo, Japan) with a 1.25X magnification objective lens (Olympus PlanApo N, Shinjuku, Tokyo, Japan) and interacted with EOS Utility software (Canon U.S.A., Inc, Melville, NY, USA). During the image capturing, a filter with an excitation wavelength of 480 nm and an emission wavelength of 535 nm was used for the inputted FAM samples. The ISO and exposure time of the camera was set to be 6400 and 0″ 5′ to capture images with fluorescence signals. The APT stepper motor controller (Thorlabs, Newton, US) was integrated with the stage of the fluorescence microscopy, which was turned on during the whole experiment to fix the location of the device while making sure the measurements of the fluorescence intensity were from the identical channels.

The FAM dye was also diluted to 10 nM and 10 µM as the samples to evaluate the cleaning efficiency with the whole streamlined operation. The left and right TESLIs were evaluated separately. The operation started by pipetting a drop of 10 nM FAM sample onto the inlet port to be generated with one 50 nL droplet followed with subsequent air evacuation and additional pipetting of 7 µL deionized (DI) water for channel washing. Upon washing, next 10 µM FAM sample was pipetted onto the same sample inlet port to be generated with one 50 nL droplet followed with the same cleaning instructions. The same process was repeated for 5 times with additional 10 nM FAM sample to be loaded at the end. A total of 11 droplets were then flowed through the incubation channel to be detected by our custom-built laser-induced fluorescence (LIF) detector, which was equipped with a 488-nm laser source (OBIS, Coherent, Inc) for excitation and a silicon avalanche photodiode (APD) for counting the photons. The laser was operated at 1 mW power and was focused into the narrowed detection zone using a × 40 objective (Thorlabs RMS40X-PF, NA 0.75, focal depth ~ 0.6 nm). The fluorescence signal emitted from the droplets were continuously captured by the APD with 0.1-ms sampling time and recorded by the custom LABVIEW program (NI, Austin, TX, USA).

The dead volume of the sample storage channel was measured by calculating the volume difference between the loaded sample inputs and the total volume of the generated droplets. The sample storage channel was filled with DI water (~ 700 nL) as the sample, which was subsequently used to generate 50-nL droplets until the DI water was completely used. Image of each generated droplet in the droplet assembly channel was captured by the same camera setup used for evaluating the cleaning efficiency under bright field for further analysis.

### Device characterization

The microvalve opening time and the droplet volume from the reagent inlets and the sample inlets were correlated to precisely control the droplet composition and size. Two operation pressures were used with 5 psi for reagent inlets and 3.5 psi for sample inlets. For reagent inlets, DI water was pre-loaded into Tygon microbore tubing to be inserted at the designated reagent inlet ports. Triplicate droplets were generated with different microvalve opening time ranging from 0.05 to 0.35 s with a 0.05 s increment. For sample inlets, a drop of DI water was pipetted at the sample inlet port to be processed by TESLI. Triplicate droplets were generated with different microvalve opening time ranging from 0.05 to 0.3 s with a 0.05 s increment. Bright field images for each droplet were captured by the digital single-lens reflex (DSLR) camera mounted on the same fluorescence microscopy setup mentioned before.

Laser power of the LIF detector was tuned to ensure wide dynamic range to detect the various concentrations of FAM samples. Four concentrations of FAM were prepared and diluted, 10 nM, 100 nM, 1 µM, and 10 µM. TESLI was used to process these samples with 12 droplets for each concentration. Generated droplets flowed through the incubation channel and were detected by our LIF system. The laser power was tuned to be 1 mW, 2 mW, 4 mW, and 10 mW in sequence for every three droplets. The photon counts for each droplet under different laser powers were recorded by the custom LABVIEW program for further analysis in MATLAB.

### Fluorescence sample infusion and detection

Two fluorescence dyes were purchased for demonstrating the device including FAM dye and resorufin dye (Thermo Fisher Scientific, Waltham, MA, USA). 11 concentrations of FAM dyes were prepared on the bench including 0 nM (i.e., DI water), 10 nM, 20 nM, 40 nM, 100 nM, 200 nM, 400 nM, 1 µM, 2 µM, 4 µM, and 10 µM. The loading sequence of the FAM dyes were randomized to be 1 µM, 40 nM, 4 µM, 400 nM, 20 nM, 200 nM, 10 nM, 0 nM (i.e., DI water), 2 µM, 100 nM, and 10 µM to better simulate the distinct samples. One concentration of resorufin dye of 2 µM were prepared and pre-loaded into Tygon microbore tubing to be inserted into the reagent inlet port. In order to capture fluorescence signals for two fluorescence dyes with different excitation and emission wavelengths, a dual-laser induced system that was incorporated with a 488-nm and a 552-nm laser source (OBIS, Coherent, Inc) for excitation and a silicon avalanche photodiode (APD) for counting the photons was used. Before the operation, we processed the 11 FAM dyes using our TESLI to generate one 50 nL droplet for each concentration, which was detected and measured using the resorufin channel (552-nm laser) as the crosstalk signal.

The operation was pre-designed to generate a 50 nL droplet mixed with FAM dye and resorufin dye with 1:1 ratio for each concentration of the 11 FAM dyes. Each FAM dye was pipetted onto the sample inlet port, infused via a TESLI into the device, assembled into a droplet, and evacuated from the device by air, before 7 µL DI water was pipetted to clean the channels to prepare for the next sample to be loaded in. The 11 FAM dyes were processed for 4 times, which required 44 times of sample switching and channel cleaning in a continuous flow. The generated droplets were then flowed through the incubation channel and detected by LIF detector with 1 mw laser power. The photon counts for each droplet were captured by the APD with a 0.1-ms sampling time and recorded by the custom LABVIEW program.

### Data analysis

During cleaning efficiency evaluation, all the fluorescence intensity measurements were acquired via ImageJ (NIH, Bethesda, MD, USA) and analyzed via Origin (Electronic Arts, Redwood City, CA, USA). For evaluating the dead volumes of the TESLI, the total volume of the generated droplets was acquired by multiplying the droplet area obtained from ImageJ with the actual channel height measured by VK-X100K laser microscope (Keyence Corporation, Osaka, Japan). The images captured for correlation between microvalve opening time and droplet volume were also measured via ImageJ to acquire the droplet area to be multiplied with the channel height to obtain the droplet volume. The comparison, further data analysis, and plotting were done via Origin. The recorded fluorescence signal from the LABVIEW program were all analyzed via MATLAB and Excel and plotted via Origin. The distribution of the droplet size was normalized to its average and plotted via Origin.

## Results and discussion

### Overview of tubing-free sample loading and integrated droplet device

**Figure 1:**
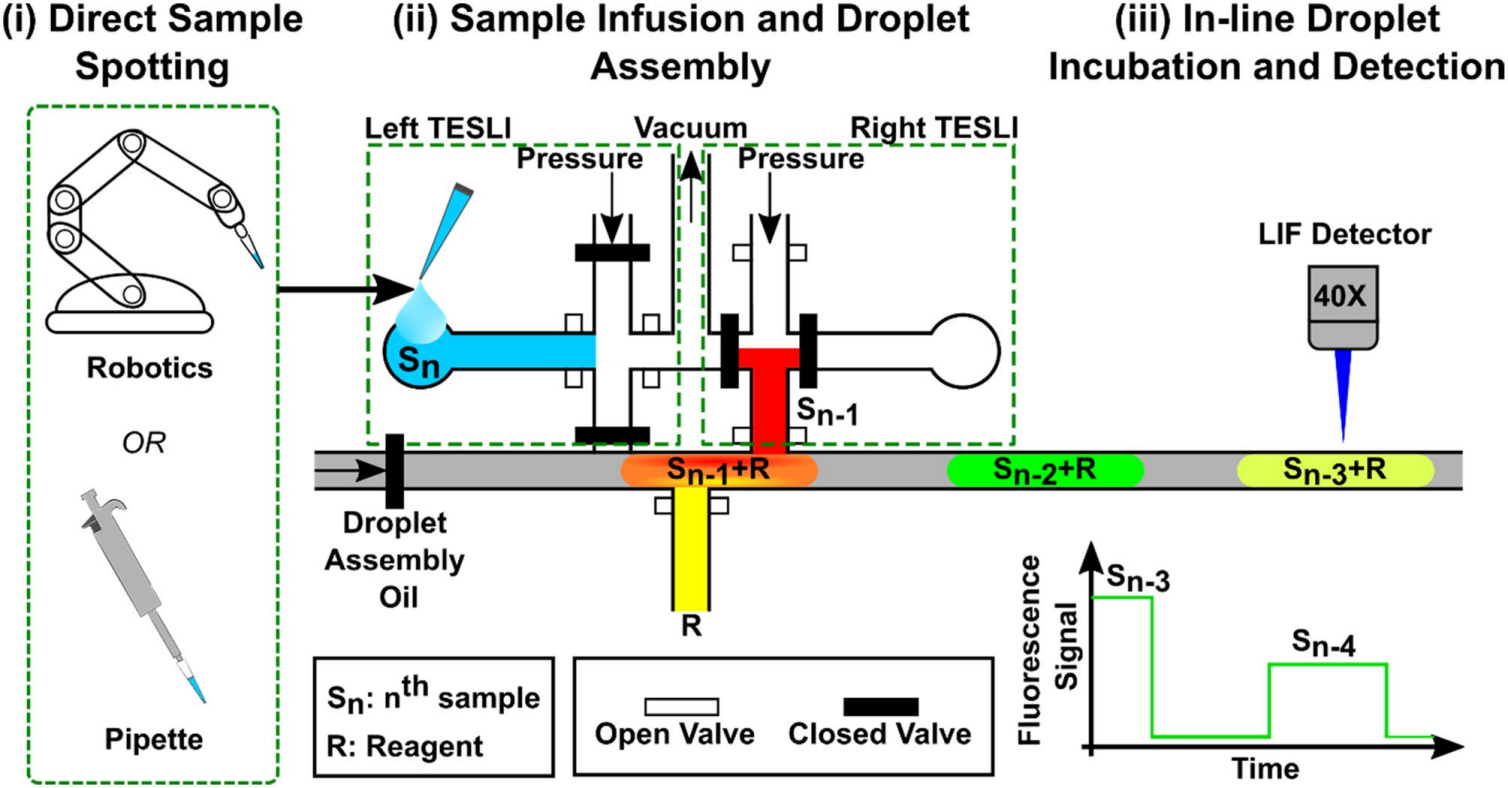
Streamlined workflow enabled by TESLI (Tubing Eliminated Sample Loading Interfaces) and integrated droplet device. The streamlined workflow is composed of (**i**) direct sample spotting, (**ii**) sample infusion and droplet generation enabled by dual TESLIs, and (**iii**) in-line droplet incubation and detection. (**i**) Both robotics and pipetting can be employed to directly spot (**ii**) a new sample (S_n_) onto an open-to-atmosphere inlet port of TESLI of the integrated droplet device. As S_n_ is infused into the device through the left TESLI by opening the microvalves connected to a vacuum source, the previously loaded sample (S_n−1_) in the right TESLI and a reagent (R) in a reagent channel are injected to form a nanoliter droplet by opening the microvalves connected to pressure sources. The dual TESLIs can alternate their operations, thereby achieving simultaneous sample switching and droplet generation. Once assembled, every droplet is propelled by pressurized droplet assembly oil to flow orderly downstream for (**iii**) in-line droplet incubation and in-line fluorescence detection in the same order as it is generated. As every droplet sequentially flows through the detection zone, a custom laser induced fluorescence (LIF) detector measures its emitted fluorescence signal, resulting in a fluorescence trace from all droplets as a function of time for visualization and analysis.

The development of TESLI and the integrated droplet device facilitates a streamlined workflow of direct sample spotting, TESLI-based sample infusion and droplet generation, and in-line droplet incubation and detection (Fig. 1). Rather than preloading the samples in tubings and then inserting the tubings into inlet ports, samples can be directly spotted over the open-to-atmosphere inlet ports of TESLIs via either robotics or pipetting (Fig. 1(i)) before they are infused into the device. Such direct sample spotting is possible because TESLI uses a network of programmable pneumatic microvalves to first regulate a vacuum source for infusing the spotted sample into the device and then regulate a pressure source for generating droplets from the infused sample. Moreover, a pair of TESLIs are incorporated in the same device to alternate operation and minimize idle time (Fig. 1(ii)). While a newly spotted sample (S_n_) is infused through the inlet port of one TESLI into the device via vacuum, a previously loaded sample (S_n−1_) in the other TELSI is concurrently pressurized into the oil-filled droplet assembly channel and mixed with the reagent (R) that is simultaneously pressurized from the reagent channel into the droplet assembly channel to form a nanoliter droplet (Fig. 1(ii)). Every generated droplet is then propelled downstream by the pressurized oil phase for in-line incubation with a fixed position relative to adjacent droplets such that the droplet position functions as a unique barcode (Fig. 1(iii)). Finally, as every droplet sequentially flows through the detection zone in the device, a custom laser induced fluorescence (LIF) detector measures the fluorescence signal from every droplet, resulting in a fluorescence trace for analysis (Fig. 1(iii)).

**Figure 2:**
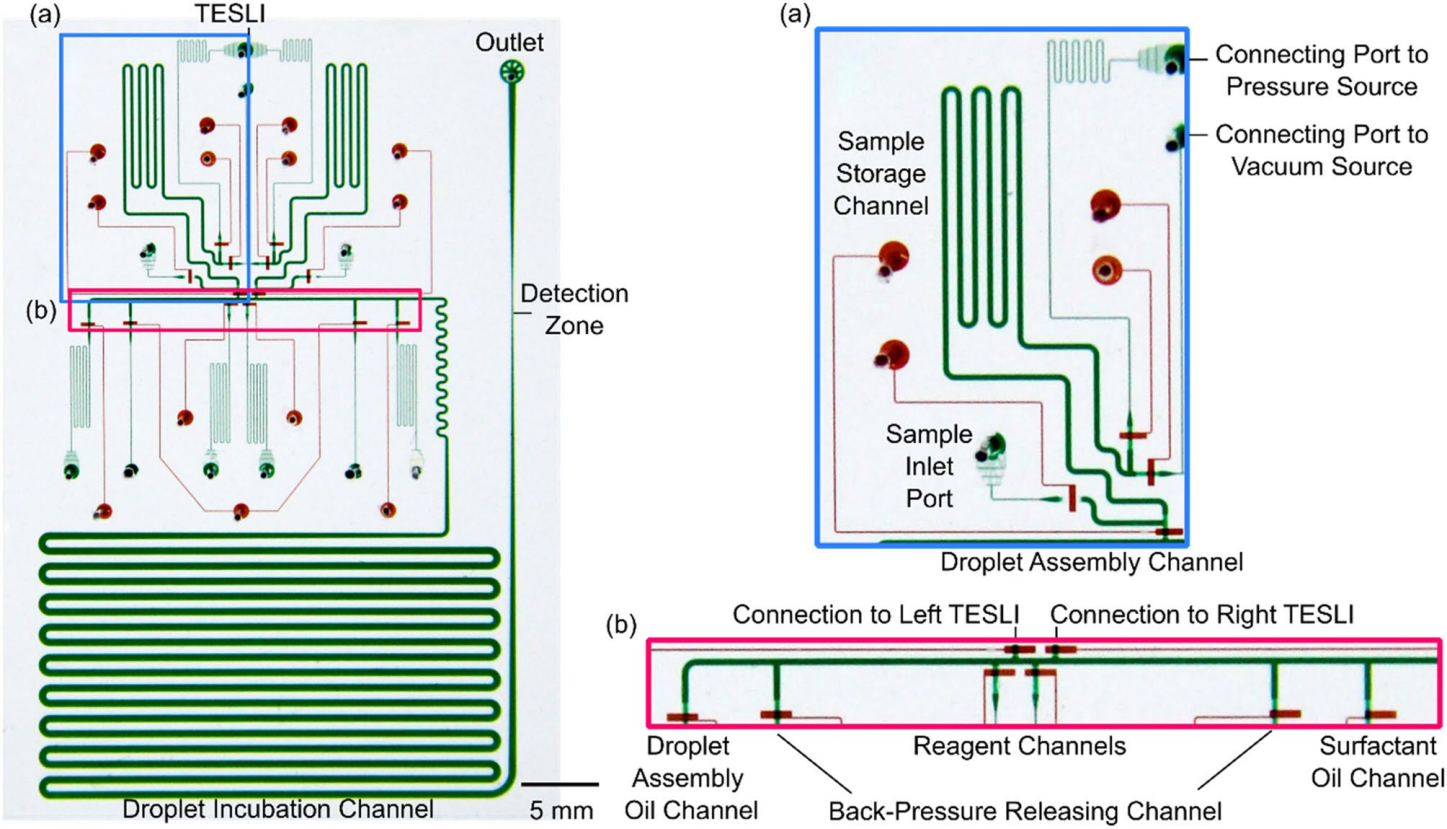
Integrated droplet device with dual TESLIs. The integrated droplet device is composed of two PDMS layers: the top fluidics layer where droplets are generated, incubated, and detected (filled with green dye for visualization) and the bottom valve layer where microvalves are actuated to regulate droplet generation and flow (filled with red dye for visualization). (**a**) Each TESLI is composed of an open-to-atmosphere sample inlet port for directly spotting samples, a sample storage channel for storing the infused samples, a connecting port to an external vacuum source for infusing samples, a connecting port to an external pressure source for injecting infused samples into droplets, and four corresponding microvalves for controlling the fluid flow. (**b**) Droplet assembly is conducted in the 200-µm wide droplet assembly channel, to which the inlet nozzles from dual TESLIs and the inlet nozzles of two reagent channels are connected. Upstream from droplet assembly channel is the droplet assembly oil inlet for introducing the oil phase that facilitates droplet assembly and propels generated droplets downstream. Two back-pressure releasing channels that flank the droplet assembly channel can open to atmosphere, alleviate build-up of back-pressure, and ensure volume uniformity of assembled droplets. Further downstream to the droplet assembly channel is the surfactant oil treatment inlet for introducing the surfactant-added oil phase that treats the 500-µm-wide incubation channel downstream and prevents droplets from sticking to the incubation channel. The end of the incubation channel narrows to a 100-µm-wide detection zone, where the custom LIF detector is aligned to for detecting the fluorescence signals from droplets.

For demonstration, we designed an integrated droplet device with dual TESLIs and subsequently microfabricated it via multi-layer PDMS soft lithography^40^ (Fig. 2). Similar to our previous devices^41–45^, this device adopts the two-layer architecture with the bottom valve layer (Fig. 2, red) housing “push-up” microvalves^41,44–46^ to regulate the fluid flow in the top fluidics layer (Fig. 2, green). Every push-up microvalve contains a thin membrane that is sandwiched between the two layers and is programmatically controlled by an external pressure source—the microvalve is closed when the membrane is deflected upward to stop fluid flow in the top fluidics layer when external pressure is turned on and the microvalve is open when the membrane relaxes to allow fluid flow in the top fluidics layer when external pressure is turned off (Fig. S1). A network of such microvalves, each individually programmable, are integrated to enable TESLI. Specifically, TESLI is composed of a sample inlet port that is open to atmosphere to allow direct sample spotting, a sample storage channel that can store 700 nL of each infused sample, a connecting port to an external vacuum source, and a connecting port to an external pressure source—all of which are regulated by corresponding microvalves (Fig. 2a). Moreover, a scalable design for dual TESLIs is accomplished by joining their connecting ports so that they share the same external vacuum source and external pressure source. In this device, the 200-µm-wide droplet assembly channel, which connects the inlet nozzles of both TESLIs and the inlet nozzles of two reagent channels, is where samples and reagents are injected for assembling droplets (Fig. 2b). The droplet assembly oil inlet located upstream to the droplet assembly channel serves to introduce the oil that facilitates droplet assembly and propels all generated droplets downstream. The two back-pressure releasing outlets flanking the droplet assembly channel help maintain consistent droplet volume by opening to atmosphere during droplet generation and reducing back-pressure. The surfactant oil channel located downstream to the droplet assembly channel is used to introduce the oil that pre-treats the 500-µm-wide incubation channel and prevents droplet sticking. Finally, a 100-µm-wide detection zone is positioned at the end of the incubation channel. The narrow cross-section of the detection zone serves to elongate droplets as they flow through, which allows the LIF detector to collect more data points from every droplet. Using this continuous flow design, this device can therefore generate, incubate, and detect droplets in an assembly line-like workflow.

### Operation of TESLI

**Figure 3:**
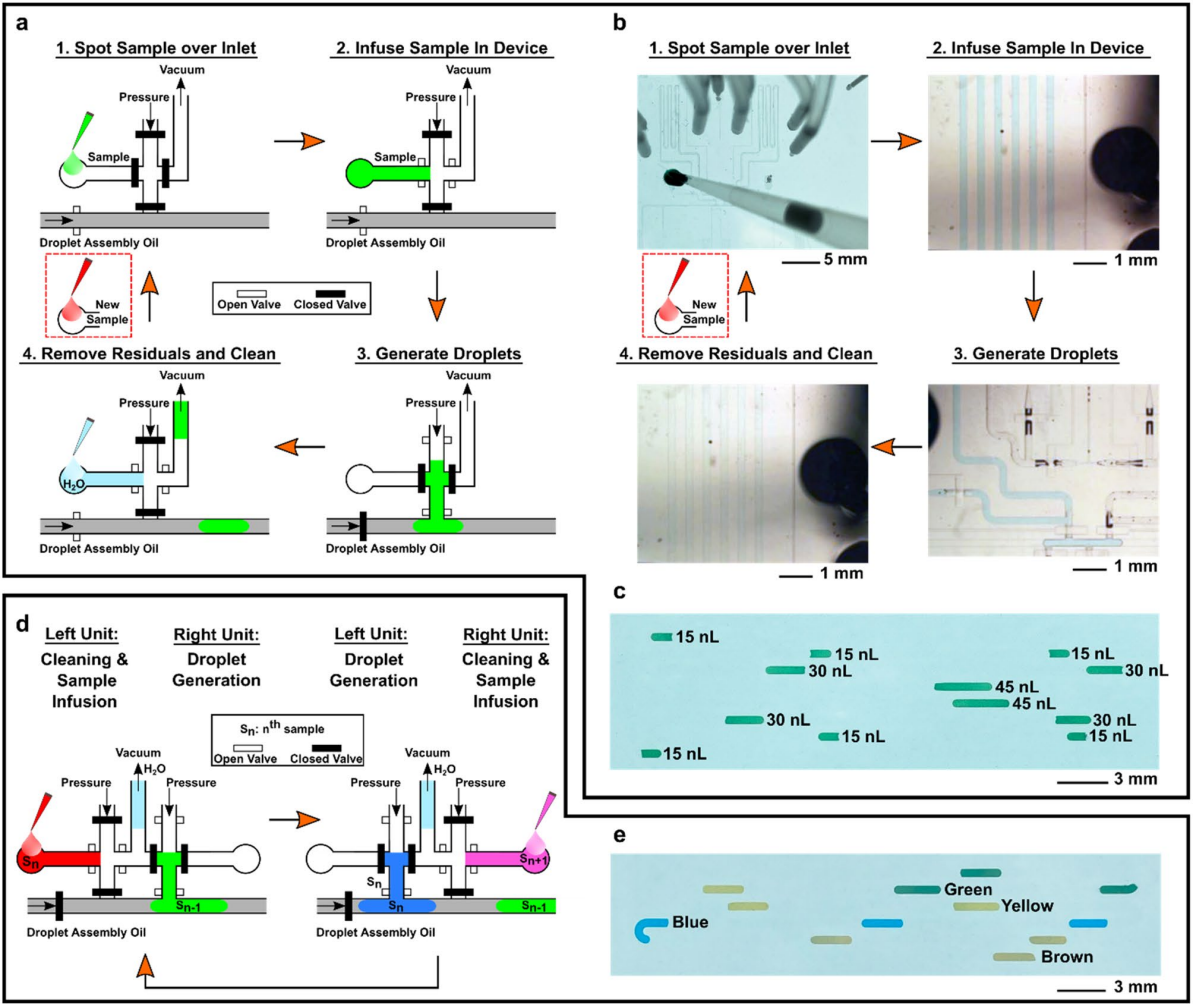
Operation of TESLI. (**a**) TESLI operates in a 4-step cycle (shown here in a schematic): 1. Spotting sample over inlet, 2. Infusing sample in device, 3. Generate droplets, and 4. Removing residuals and cleaning. (**b**) The 4-step operation is demonstrated in an integrated droplet device using blue food dye as the sample via photography (step 1) and bright-field microscopy (steps 2–4), which focuses around either the sample storage channel (steps 2 and 4) or the droplet assembly channel (step 3). (**c**) Using green food dye as the sample, programmable droplet generation upon sample loading using TESLI is demonstrated through a series of droplets with the programed order of 3–15 nL droplets, 2–30 nL droplets, 1–45 nL droplet followed by the reversed order of 1–45 nL droplet, 2–30 nL droplets, and 3–15 nL droplets. (**d**) When two TESLIs are paired to alternate droplet generation with sample infusion and cleaning, samples can be processed at scale without adding idle time. (**e**) As demonstration, brown, blue, yellow, and green food dyes are infused by the dual TESLIs into the integrated droplet device, where each dye is used to generate 3–30 nL droplets, which maintain the same order as sample infusion in the incubation channel. Moreover, uniform spacing between droplets generated from different dyes under fixed microvalve opening times indicates that the samples are infused, and the droplets are generated without idle time.

TESLI operates in a 4-step cycle: spotting sample over the inlet, infusing and storing sample in the device, generating droplets from the infused sample, and cleaning any unused sample in the device (Fig. 3a). For each new sample, TESLI simply repeats the cycle, thereby achieving scalable operation. Using a blue food dye as the sample, we demonstrated this 4-step operation (Fig. 3b and Videos S1 and S2). We first spotted 700 nL drop of the sample over the inlet (Fig. 3b(i)). We then activated vacuum to infuse the sample into the sample storage channel of the device (Fig. 3b(ii)). We next activated pressure under a pre-programmed sequence to generate a nanoliter droplet into the main droplet assembly channel of the device (Fig. 3b(iii)). Finally, we activated vacuum to remove unused sample and clean the sample storage channel with air and then DI water spotted over the inlet (Fig. 3b(iv)), readying TESLI for the next sample. Importantly, we note that only sample and DI water spotting were manual, the rest of TESLI operation were automated. As previously noted, TESLI can generate droplets with preprogramed volumes from each infused sample. As demonstration, we used TESLI to infuse a green food dye as the sample and generate a series of droplets with various volumes and replicates—3 replicates of ~ 15 nL droplets, 2 replicates of ~ 30 nL droplets, and 1–45 nL droplet followed by the reversed order of 1–45 nL droplet, 2 replicates of ~ 30 nL droplets, and 3 replicates of ~ 15 nL droplets (Fig. 3c).

Multiple TESLIs can be combined for further enhancement. For example, two identical TESLIs can be paired to alternate operation—while one TESLI finishes cleaning and infuses a sample, the other TESLI generates droplets from an already infused sample, and vice versa (Fig. 3d). As the two TESLIs alternate operation for every new sample, unnecessary idle time from cleaning and sample switching can be avoided, thereby improving the throughput of the device. For demonstration, we used such dual TESLIs to infuse 4 food dyes (brown, blue, yellow, and green) into our device and generate 3–30 nL droplets from each food dye in the device (Fig. 3e). The alternating operation of dual TESLIs enabled the infusion of these 4 samples and the production of these droplets in the same order as sample infusion. Moreover, as we programmed fixed microvalve opening times for generating droplets and propelling them downstream, uniform spacing between the droplets generated from different dyes indicates that the samples were infused, and the droplets were generated without idle time. These results thus demonstrate that dual TESLIs indeed achieved scalable sample loading while avoiding idle time.

### Characterization of cleaning efficiency and dead volume

**Figure 4:**
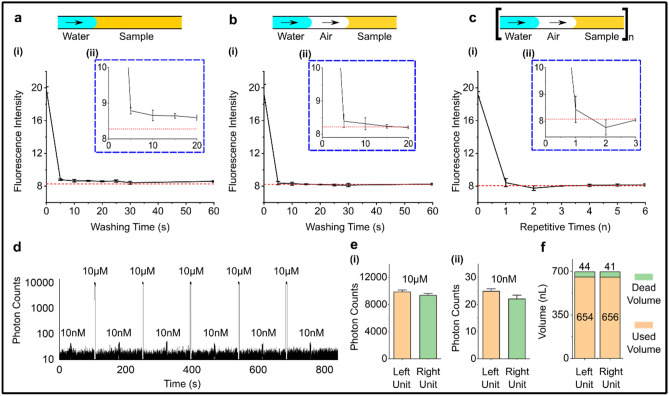
Characterization of cleaning efficiency and dead volume. Three cleaning protocols—(**a**) direct continuous DI water wash, (**b**) air evacuation before continuous DI water wash, and (**c**) cyclic air evacuation and DI water wash—are evaluated after a fluorescence dye (FAM) is loaded into a TESLI sample storage channel. (**i**) Fluorescence signals in the sample storage channel between FAM (i.e., channel intensity at time 0) and water (i.e., baseline intensity; red dash line) as a function of washing time/repetition for the three cleaning protocols show that the second and the third protocols provide more effective cleaning, which is evident through the (**ii**) inset plots. (**d**) Effective cleaning via air evacuation before continuous water wash is additionally demonstrated by alternate loading of 10 nM FAM and 10 µM FAM through a single TESLI with this cleaning protocol in between. Droplet generation from each FAM dye and subsequent fluorescence detection of each droplet by the custom LIF detector yield a fluorescence trace that reveals alternating weak and strong fluorescence signals corresponding to 10 nM and 10 µM FAM, which can only be observed if 10 µM FAM is effectively washed. (**e**) Both the left TESLI and the right TESLI achieve comparable cleaning efficiencies. (**f**) The dead volumes—measured by the difference between a fixed sample volume (700 nL here) and the used volume (i.e., total volume of the droplets generated from the sample)—of the left and the right TESLIs are 44 nL and 41 nL, respectively. In (**a**), (**b**), (**c**), and (**e**), the error bars depict ± 1 standard deviation.

Because we used each TESLI to sequentially infuse multiple samples, the risk for cross-contamination must be minimized through effective cleaning. To this end, we evaluated 3 cleaning protocols—direct continuous DI water wash (Fig. 4a), air evacuation followed by continuous DI water wash (Fig. 4b), and cyclic air evacuation and brief DI water wash (Fig. 4c). To do so, we first infused DI water into the sample storage channel and used fluorescence microscopy to acquire an image of the sample storage channel and measure the baseline fluorescence intensity (Fig. 4a–c, red dashed lines, and Fig. S2). We then infused FAM (the most used green fluorescent dye) as the sample into the sample storage channel and measured the sample fluorescence intensity before initiating one of the three cleaning protocols and measuring the fluorescence intensity throughout the cleaning protocol (Fig. S2). Thus, effective cleaning corresponds to the fluorescence intensity returning to the baseline. Upon initiating all 3 cleaning protocols, we observed immediate decreases of the fluorescence intensities that all gradually approached the baseline, indicating effective cleaning (Fig. 4a–c). However, to minimize cross-contamination, it was necessary to compare minute increases in fluorescence intensity (Fig. 4a–c, zoom-in figure). The fluorescence intensities from the last two cleaning protocols returned to the baseline intensity whereas the fluorescence intensity from the first cleaning protocol remained above the baseline intensity, which suggests uncleaned FAM residue remaining in the sample storage channel. Between the last two cleaning protocols with comparably effective cleaning, we selected the second cleaning protocol (i.e., air evacuation followed by continuous DI water wash) for the remainder of this work because this protocol required spotting DI water only once rather than 8 times as in the cyclic cleaning protocol.

As additional demonstration of effective cleaning via air evacuation followed by continuous DI water wash, we measured the fluorescence intensities of alternating FAM dyes that differed 1000-fold in concentrations. Here, we alternated loading of 10 nM and 10 µM FAM through the same TESLI and cleaned the TESLI via air evacuation followed by continuous DI water wash in between. For each loaded FAM dye, we generated a droplet and detected its fluorescence signal using our custom LIF detector. In the resulting fluorescence trace, we observed alternating weak and strong fluorescence signals corresponding to 10 nM and 10 µM FAM, respectively (Fig. 4d). Importantly, the fluorescence signals from all 10 nM FAM remained consistent with the first 10 nM FAM, which could only be observed if all preceding 10 µM FAM were effectively washed cleaned without leaving residue behind (Fig. 4d). Moreover, we tested both TESLIs in the device and confirmed that similar cleaning efficiencies could be achieved (Fig. 4e). These results thus provide additional support for effective cleaning of TESLI via air evacuation and continuous DI water wash.

We also measured the dead volume of TESLI to show small sample wastage. To do so, we filled the sample storage channels of dual TESLIs with ~ 700 nL DI water, and generated multiple 35-nL droplets from the DI water. The dead volume was calculated as the difference between the sum of the droplet volumes and the 700-nL input volume, which were 44 nL and 41 nL for left and right TESLI storage channels, respectively (Fig. 4f). The dead volumes predominantly resulted from a segment of the storage channel near the sample inlet port that cannot be pressurized, rendering the sample in this segment unusable for droplet generation (Fig. S3). We also note that when there was unused sample left in this segment of the storage channel, direct washing with DI water without air evacuation would merely dilute the unused sample. This observation explains why this cleaning protocol was less efficient than the other two protocols that used air to first clear unused samples (Fig. 4a–c). This segment of the storage channel, which was originally designed for properly placing the nozzles and routing the microvalves, will be minimized in future designs.

### Scalable sample loading by TESLI with droplet generation, incubation, and detection in device

Prior to demonstrating sample loading by TESLI, we calibrated our device for precise droplet generation and our LIF detector for proper fluorescence detection of our intended sample and reagent. We first calibrated the droplet volume as a function of the microvalve opening time for sample channels and reagent channels at their normal injection pressures. We found that, for sample channels, opening microvalves for 0.05–0.30 s under 3.5 psi produced 7.0 ± 0.4–50.9 ± 0.4 nL droplets with a strongly linear relationship, and for reagent channels, opening microvalves for 0.05–0.35 s under 5.0 psi produced 7.7 ± 0.1–45.5 ± 0.4 nL droplets also with a strongly linear relationship (Fig. S4A). Low coefficients of variation (CVs) of both sample droplet volume (< 6%) and reagent droplet volume (< 2%) indicate that precise droplet generation can be achieved in our device in the absence of any additional feedback control (Fig. S4B). Moreover, as we intended to use various concentrations of FAM as simulated samples and a fixed concentration of resorufin (a red fluorescent dye) as the simulated reagent in our final demonstration, we ensured that our LIF detector could properly detect both dyes. Specifically, we tuned the laser power of the LIF detector and found that 1 mW laser power allowed us to detect a wide concentration range of FAM (Fig. S5). We also measured the intensities of unwanted crosstalk signal in the resorufin channel that originated from only FAM so that any crosstalk signal in the final demonstration could be subtracted from the real resorufin fluorescence signal (Fig. S6). These calibration results thus readied us for the final demonstration.

**Figure 5:**
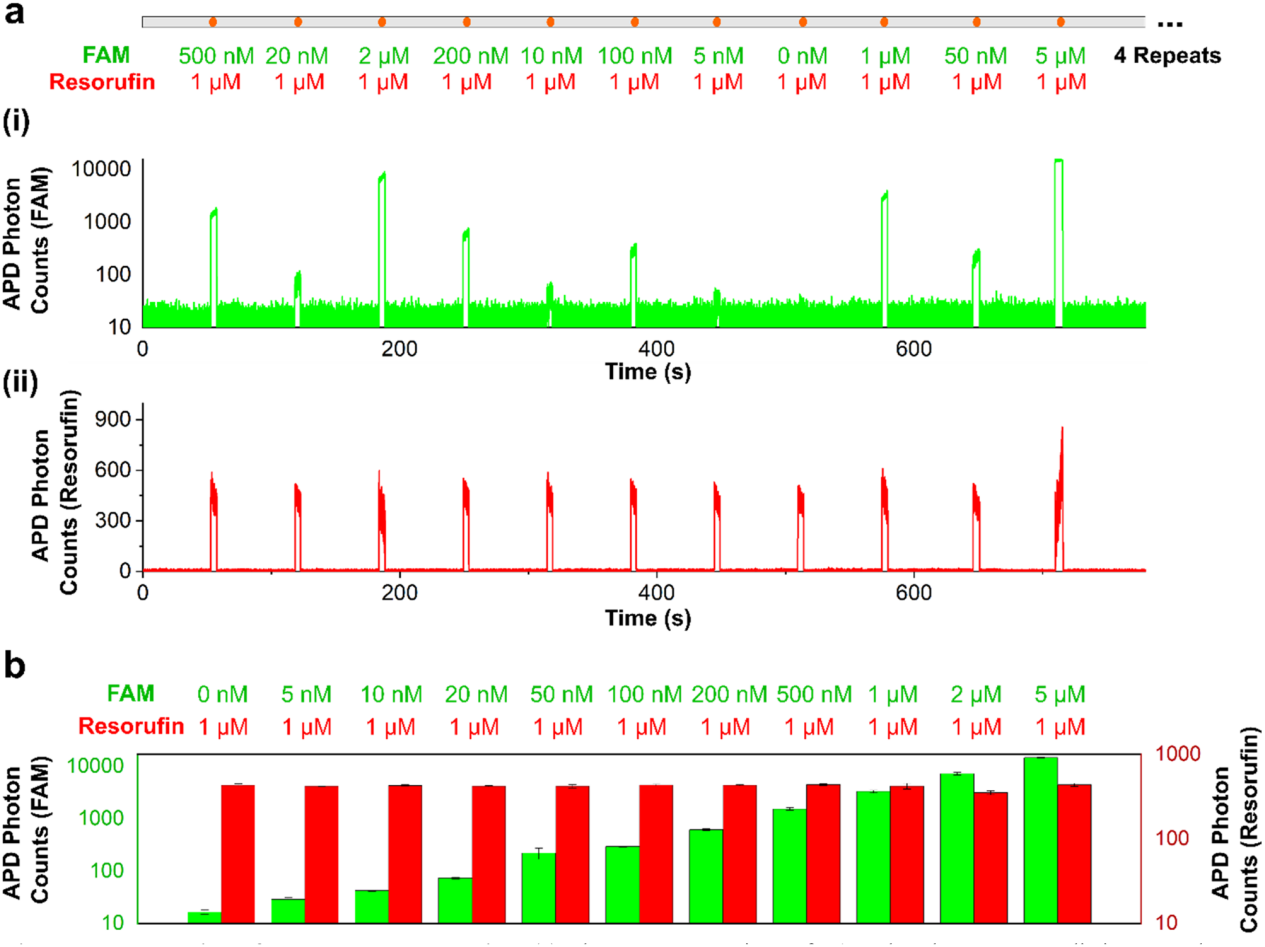
Demonstration of scalable sample loading. (**a**) Eleven concentrations of FAM dye that represent distinct samples are loaded in a scrambled sequence through dual TESLIs into the integrated droplet device and assembled with resorufin at 1:1 ratio to generate distinct droplets. To emphasize on sample loading and switching, only one droplet is generated per FAM sample. The sequence of the droplets based on their FAM final concentrations is 500 nM, 20 nM, 1 µM, 200 nM, 10 nM, 100 nM, 5 nM, 0 nM (i.e., water), 1 µM, 50 nM, and 5 µM, and all droplets contain 1 µM resorufin. The droplets are briefly incubated in the device and fluorescently detected by the custom LIF detector, which uses avalanche photodiodes (APDs) to quantify photons in the droplets. (**a**(**i**)) The APD photon counts in the FAM detection channel for the 11 droplets increase and decrease in accordance with the FAM concentration of the scrambled loading sequence, while (**a**(**ii**)) the APD photon counts in the resorufin channel for all 11 droplets are uniform (after subtracting crosstalk fluorescence signal from FAM), consistent with the uniform resorufin concentration. (**b**) The same 11 concentrations of FAM dye are repeated 4 times in the same scrambled order, and we obtained comparable results among them. The error bars depict ± 1 standard deviation.

Finally, we demonstrated facile and scalable loading of multiple samples by dual TESLIs into the integrated droplet device, along with droplet generation, incubation, and detection in the device. Here, we repeated 4 times the infusion of 11 FAM dyes at various concentrations, for a total of 44 samples. The 11 FAM dyes were in a scrambled order—1 µM, 40 nM, 4 µM, 400 nM, 20 nM, 200 nM, 10 nM, 0 nM (i.e., water), 2 µM, 100 nM, and 10 µM—to simulate distinct samples. Upon infusion, each sample along with 2 µM resorufin serving as the reagent were injected at equal volume to form a ~ 50-nL droplet. As a result, in the droplets, the FAM concentrations were halved and the resorufin concentration was a constant 1 µM. Of note, here we emphasized on sample loading and therefore generated only one droplet with a fixed reagent concentration per sample, but multiple droplets with various reagent concentrations can be readily achieved, as we demonstrated through food dyes (Fig. 3) and in our earlier works^41,42,44,47^. Upon in-line incubation and fluorescence detection, in the resulting fluorescence trace, we observed droplets with distinct FAM intensities that corresponded with the order of sample infusion (Fig. 5a(i)). Also, in the resulting fluorescence trace, we observed a uniform resorufin intensity across all droplets (Fig. 5a(ii)). For the 4 replicates, we acquired consistent FAM and resorufin intensities with small standard deviations (Fig. 5b). Moreover, across the 44 droplets, we measured consistent sizes with a low CV of 5.67% (Fig. S7). These results provide strong evidence that we achieved successful infusion of 44 samples with minimal cross-contamination between successive samples, as well as reliable droplet generation, incubation, and detection.

## Conclusions

In this work, we introduce TESLI as a new means to achieving facile and scalable sample loading for droplet microfluidics with low sample wastage, zero idle time, and potential for full automation. TESLI integrates a network of programmable pneumatic microvalves that regulate vacuum and pressure sources so that successive sub-microliter samples can be directly spotted onto the open-to-atmosphere TESLI inlet, vacuumed into the device, and pressurized into nanoliter droplets within the device with minimal wastage. The same vacuum and pressure regulation also endows TESLI with cleaning and sample switching capabilities, thus enabling scalable processing of many samples in succession. In this work, we further incorporated two TESLIs in an integrated droplet device to alternate droplet generation with cleaning and sample switching and minimize unnecessary idle time, while the integrated droplet device enables in-line droplet incubation and detection in an assembly line-like workflow. For minimizing cross-contamination between successive samples in TESLI, we evaluated cleaning protocols and found that air evacuation of unused sample followed by DI water wash provided effective yet simple cleaning. We also showed that the dead volume of TESLI was in the nanoliter range. As demonstration, we used our integrated droplet device with dual TESLIs to process and analyze 44 samples, which not only represents the cutting edge for existing droplet microfluidics but also has the capacity to scale. Our work thus demonstrates that tubing-, reservoir-, or tank-free sample loading for droplet microfluidics is not only feasible but also potentially promising.

Despite these promising initial results, our TESLI and integrated droplet device have limitations and thus require future improvements. First, we currently manually pipette samples and DI water, but we envision leveraging unique open-to-atmosphere sample spotting capability of TESLI and incorporating pipetting robotics such as OpenLH^48^ and other open-source pipetting systems^49^ to achieve full automation. We also plan to design a new TESLI that can perform cleaning without repeated spotting of DI water even by pipetting robotics. Second, we combined TESLI with pneumatic microvalve-based droplet generator, which to date had demonstrated hundreds of nL-scale droplets per device, but we foresee that TESLI can be flexibly combined with different droplet generators^14^. In particular, we plan to combine TESLI with our previous integrated Programmable Picodroplet Assembler (iPPA)^44^—a device that can generate tens of unique groups of thousands of droplets by integrating pneumatic microvalve-based droplet generator and flow-focusing junction-based droplet generator. This combined device could achieve unprecedented scalability for droplet microfluidics. Third, we demonstrated pairing two TESLIs, but we see the possibility of implementing more than two TESLIs per device. We note that, however, each additional TESLI would require 4 more microvalves. Finally, we used FAM as simulated sample for demonstration, but we see different chemical and biological assays to be implemented in our device, including matrix metalloproteinase (MMP) screening assay^42^, cytochrome P450 screening assay^50^, luminescent nanomaterial cell screening assay^51^, other inhibitor and drug screening assays^52–54^, and bacteria detection and antimicrobial susceptibility testing assay^45^. With these future improvements, TESLI has the potential to become a powerful sample loading approach in droplet microfluidics for a variety of analytical and bioanalytical applications.

## Supplementary Information

Supplementary Information 1.

Supplementary Video 1.

Supplementary Video 2.

## Data Availability

The datasets generated during and/or analyzed during the current study are available from the corresponding author on reasonable request.
