# Supplementary material for: Facile and scalable tubing-free sample loading for droplet microfluidics
Source: Sci Rep. 2022 Aug 3;12:13340. doi: 10.1038/s41598-022-17352-3 (PMC9349288; doi:10.1038/s41598-022-17352-3)
Supplement: Supplementary file 1 — Supplementary Information 1. [file 41598_2022_17352_MOESM1_ESM.docx]

**Electronic Supplementary Information (ESI)**

*for*

Facile and Scalable Tubing-Free Sample loading for Droplet Microfluidics

Fangchi Shao,^a^† Kuangwen Hsieh, ^b^† Pengfei Zhang, ^a^ Aniruddha M. Kaushik ^b^ and Tza-Huei Wang *^ab^

^a^ Department of Biomedical Engineering, Johns Hopkins University, 3400 N. Charles Street, Baltimore, MD, 21218, USA

^b^ Department of Mechanical Engineering, Johns Hopkins University, 3400 N. Charles Street, Baltimore, MD, 21218, USA

* Corresponding Author. E-mail: [thwang@jhu.edu](mailto:thwang@jhu.edu)

† The authors contributed equally.


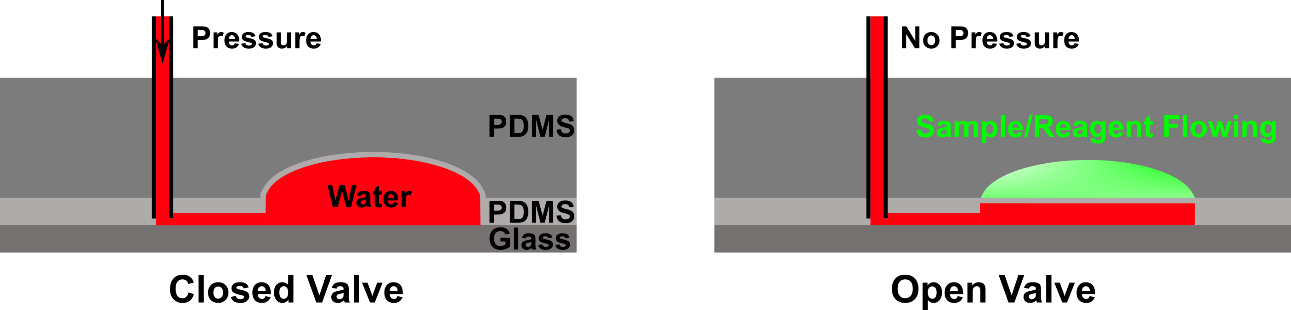


**Supplementary Fig. S1.** Schematic of the microvalves. The microfluidic is composed with two layers, a top PDMS fluidic layer, and a bottom PDMS valve layer. The fluidic channel above the microvalves is fabricated with rounded cross-section for better sealing. When the microvalve is closed, the pressure source is turned on to push water to deflect the thin PDMS membrane to seal the top fluidic layer. When the microvalve is opened, the pressure source is turned off and the membrane relaxes for fluidic flow.


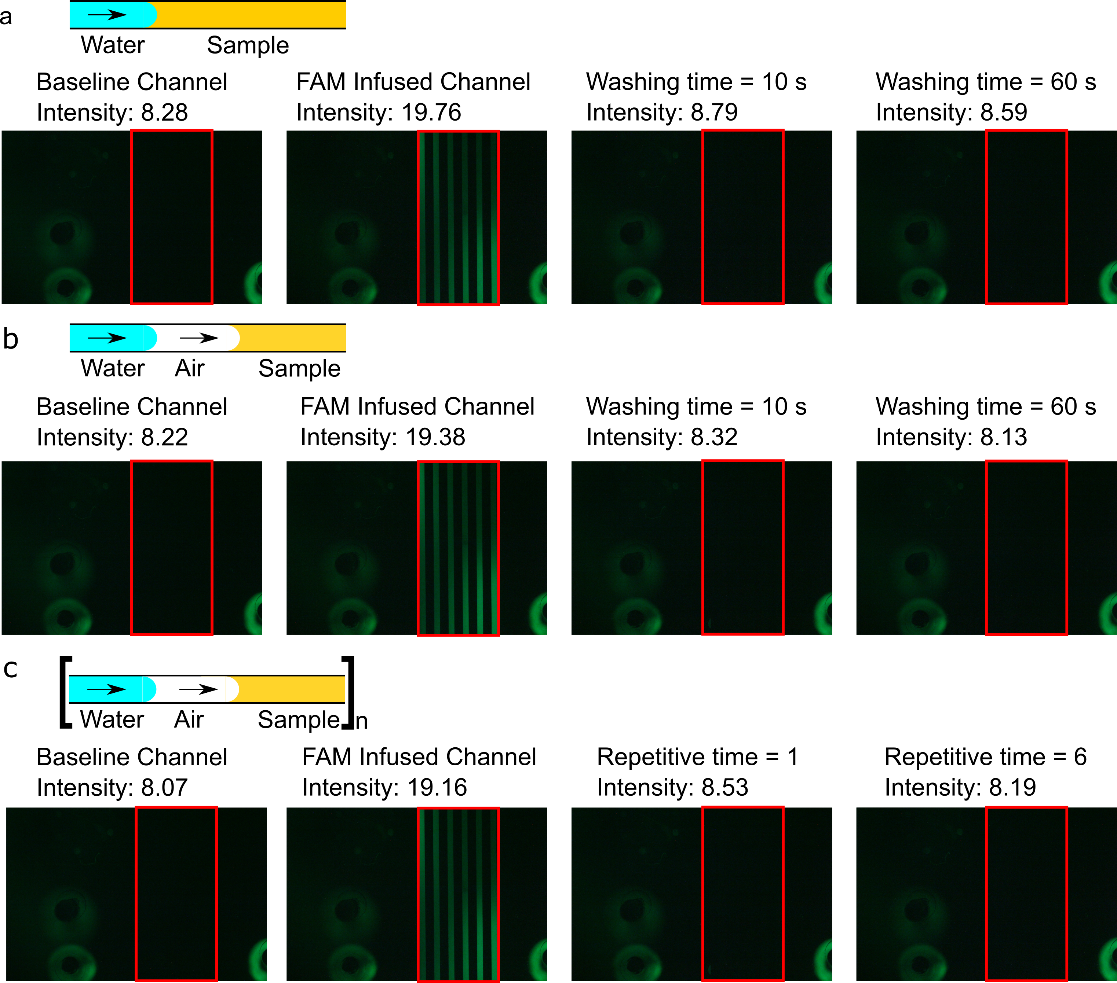


**Supplementary Fig. S2.** Examples of the acquired fluorescence images of the TESLI sample storage channel for the three-washing protocol, (a) continuous water washing, (b) air evacuation before continuous water washing, and (c) repetitive air rinsing and water rinsing. For each protocol, a baseline channel intensity of water-filled sample storage channel is obtained first before infusing the FAM sample. Two representative fluorescence images at different timepoints (a,b) or with different repetitive times (c) were showed along with the fluorescence intensity. The baseline intensity of each method is referred as the red dotted line in Fig. 4 (a), 4(b), and 4(c).


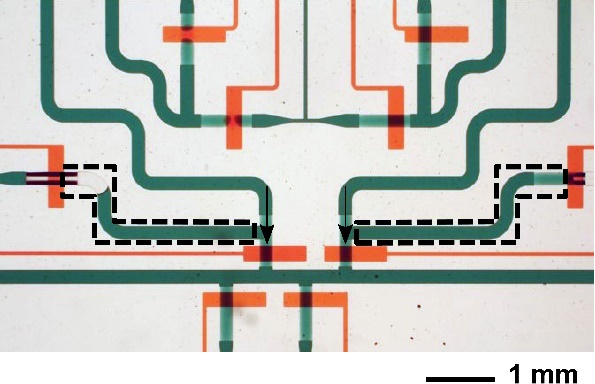


**Supplementary Fig. S3**. The dead volume comes from the design of the chip, where the dashed line enclosed the dead volume region. This region cannot be pressurized by the pressure source.


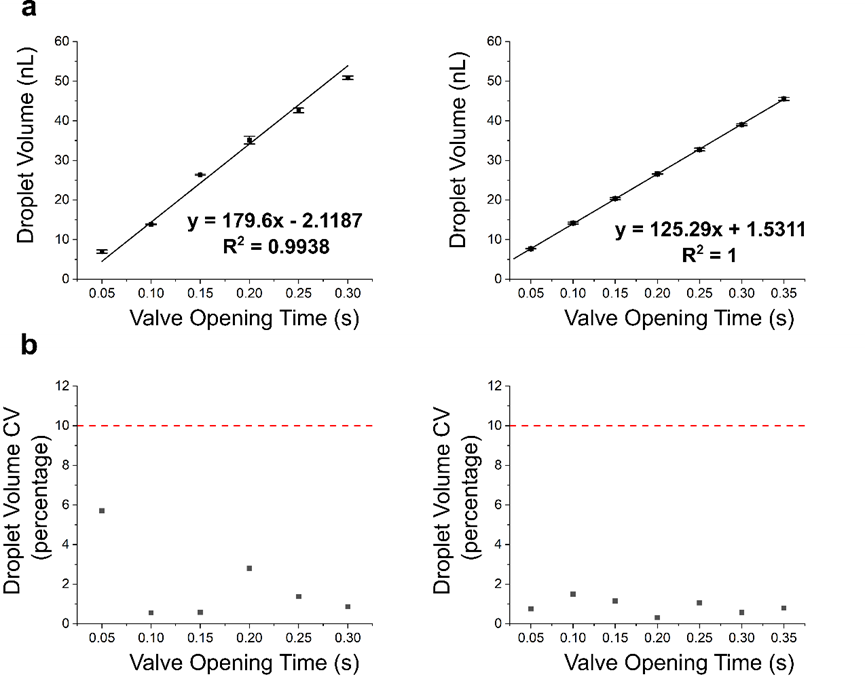


**Supplementary Fig. S4.** (a) The linear correlation between the valve opening time and the generated droplet volume at 3.5 psi (left) for pressurizing stored samples and 5 psi for pressurizing reagents (right). Different pressures were used for pushing the reagents and samples due to different back pressure from the channels. (b) The droplet volume CVs at different valve opening time. The low CVs validated the high droplet uniformity. The error bars depict ±1 standard deviation.


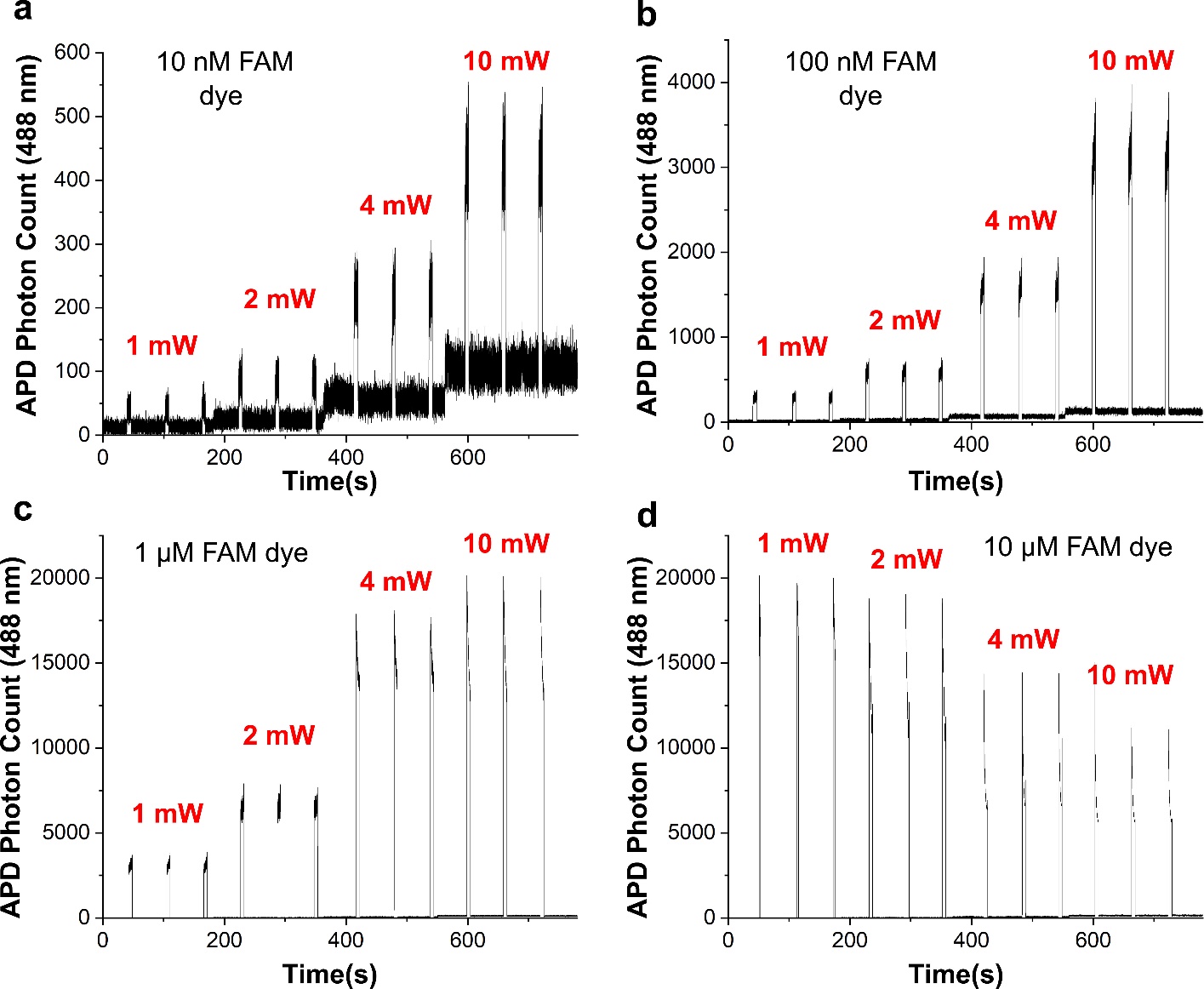


**Supplementary Fig. S5.** Dynamic range of the LIF detection system with four laser powers, 1W, 2W, 4W, and 10W. Four concentrations of FAM were tested with the various laser powers including 10nM (a), 100nM (b), 1µM (c), and 10µM (d).


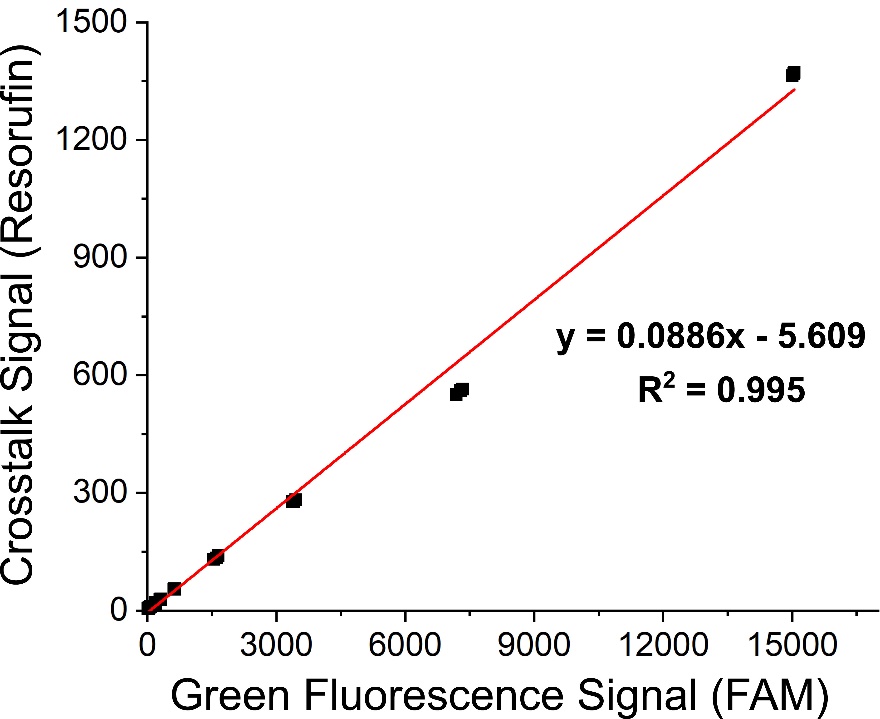


**Supplementary Fig. S6.** The linear correlation between the FAM fluorescence signal and the Resorufin crosstalk signal with triplicates (n=3).


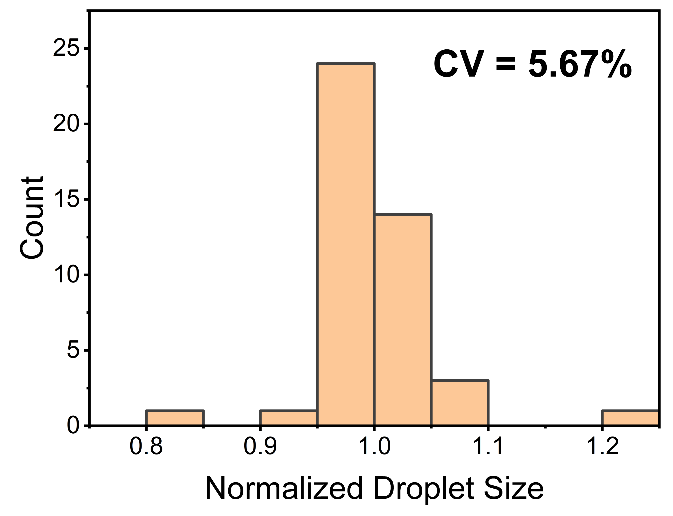


**Supplementary Fig. S7.** The normalized droplet size distribution of the 44 generated droplets in Fig. 5. The CV among the four replicates of droplets was found to be 5.67%.

**Supplementary Videos**

**Video S1. Sample Spotting Operation**. For each sample, the sample is directly spotted onto the sample inlet of the chip and infused into the sample storage channel via vacuum. For every new sample to be loaded in, cleaning steps are initiated with air evacuation and spotting of 7 µL of water to clean the unused sample in the device.

**Video S2. Parallelized Operation Procedure**. When the first sample is loaded into the sample storage channel of the right TESLI, sample droplets, reagent droplets, mixture of sample and reagent droplets are generated (Sample: Food dye; Reagent: water). After the droplet generation, the right TESLI starts the cleaning steps, while the left TESLI loads a new sample and starts the droplet generation simultaneously. The same parallelized operation repeats for every new sample to be loaded into the device.
